# Supplementary material for: Charge fluctuations in the intermediate-valence ground state of SmCoIn5
Source: Commun Phys. 2023 Aug 22;6(1):223. doi: 10.1038/s42005-023-01339-1 (PMC11041663; doi:10.1038/s42005-023-01339-1)
Supplement: Supplementary file 2 — Supplementary Information [file 42005_2023_1339_MOESM2_ESM.pdf]

Charge fluctuations in the intermediate-valence ground state of $SmCoIn_5$

David W. Tam,[1,*] Nicola Colonna,[1,2] Neeraj Kumar,[3] Cinthia Piamonteze,[4] Fatima Alarab,[4] Vladimir N. Strocov,[4] Antonio Cervellino,[4] Tom Fennell,[1] Dariusz Jakub Gawryluk,[5] Ekaterina Pomjakushina,[5] Y. Soh,[3] and Michel Kenzelmann[1,†]

[1]*Laboratory for Neutron Scattering and Imaging, Paul Scherrer Institut, 5232 Villigen, Switzerland*
[2]*National Center for Computational Design and Discovery of Novel Materials (MARVEL), Ecole Polytechnique Fédérale de Lausanne, 1015 Lausanne, Switzerland*
[3]*Paul Scherrer Institut, 5232 Villigen, Switzerland*
[4]*Photon Science Division, Paul Scherrer Institut, 5232 Villigen, Switzerland*
[5]*Laboratory for Multiscale Materials Experiments, Paul Scherrer Institute, 5232 Villigen, Switzerland*

(Dated: August 2, 2023)

SUPPLEMENTARY INFORMATION

Supplementary Note 1: Crystal field eigenstates and Coulomb repulsion in Sm^{3+}

As discussed in the main text, Sm^{3+} is a Kramers ion in the $J \approx 5/2$ spin-orbit manifold. In Supplementary Figure 1, we study the effect of the Coulomb repulsion on the eigenstates of Sm^{3+}. In Supplementary Figure 1(a), we show the evolution of the spin-orbit multiplets as the Coulomb interaction U is applied, up to the final values that we find from XLD measurements shown in the main text. It is clear that the Coulomb repulsion has a very large effect on mixing the eigenstates. The levels at the far right-hand side of the plot, corresponding to the physically realistic parameters, are shown schematically in Fig. 2(d) of the main text.

In Supplementary Figure 1(b-c), we show the role of the Coulomb interaction in the lowest $J \approx 5/2$ manifold, which represent the three Kramers doublets with wavefunctions shown in Fig. 2(j) in the main text. With no Coulomb interaction, shown at the left side of Supplementary Figure 1(b), we find that the Γ_6 eigenstate is the ground state of Sm^{3+} under the crystal field parameters we discovered. This level is separated only slightly from the Γ_7 doublet which together with the Γ_6 doublet would form the Γ_8 quartet of states under cubic O_h point group symmetry [1]. This level scheme is reversed upon applying the Coulomb interaction up to 100% of its experimental value, shown at the right side of Supplementary Figure 1(b). In Supplementary Figure 1(c), we zoom in on the level crossings near $U \sim 0.5$, which shows that the Γ_6 level crosses the two Γ_7 levels. On the other hand, the two Γ_7 levels repel each other without crossing, which is expected because they share the same irreducible representation. Therefore, we are confident that the ground state of Sm^{3+} is one of the Γ_7 doublets and that the Coulomb interaction is crucial in deciding the level scheme.

To better clarify how the eigenstates of Sm^{3+} under the total Hamiltonian (SOC+CEF+U) are different from those in the SOC+CEF approximation, in Supplementary Figure 1(d) we show the expectation value of the number operator $< N >$ for the computed wavefunction under the different symmetry components of the D_{4h} point group. Here α is the mixing parameter which couples the $J_z = 5/2$ and $J_z = 3/2$ spin-orbit eigenstates together, as is required to construct the irreducible representations Γ_6, $\Gamma_7^{(-)}$, and $\Gamma_7^{(+)}$ in the case of Ce^{3+} $(4f^1)$. Specifically, α is defined by $\Gamma_7 = \sqrt{1-\alpha^2}\,|\pm 5/2\rangle - \alpha\,|\mp 3/2\rangle$ or $\Gamma_7 = \alpha\,|\pm 5/2\rangle + \sqrt{1-\alpha^2}\,|\mp 3/2\rangle$ (Ref. [2, 3]). In Supplementary Figure 1(d), we vary α and plot the difference between the resulting $\langle N \rangle$ and the values of $\langle N \rangle$ with the Coulomb interaction applied. We find by inspection that the ground state of Sm^{3+} is near $\alpha = 0.68$, marked by a vertical grey line, but with significant differences in the A_{2u} and E_{2u} symmetry components. This indicates that the Coulomb interaction mixes the ground state wavefunction in a way that is incompatible with a single choice of α.

Supplementary Note 2: Density of states of Sm in $SmCoIn_5$

In Supplementary Figure 2, we show the calculated density of states for the Sm^{3+} and Sm^{2+} configurations using the methods described in the main text. For Sm^{3+}, shown in Supplementary Figure 2(a), we observe levels near $E - E_F$=-6 eV, consistent with the resonant ARPES results shown in Fig. 5 of the main text. We also find Sm^{3+} spectral weight near the Fermi energy, about 6 eV above the lower band, which we attribute to an upper Hubbard band given that the on-site U interaction is near 6 eV. We find therefore that this upper Hubbard band, consisting of an additional electron on the Sm site, represents a Sm^{2+} configuration and shows that the Sm^{2+} states should appear close to E_F in a mixed valence system. To confirm the stable energy of the Sm^{2+} configuration alone, we repeated the same calculations for a pure Sm^{2+} state, shown in Supplementary Figure 2(b). Here we find that the lowest band of Sm states is near $E - E_F$=-0.5 eV, which is in close agreement with the value ~ 0 found in the Sm^{3+} calculation considering the complexity of the calculations in general and especially the fact that they do not accommodate any many-body effects. Therefore, the DFT calculations give us a high degree of confidence that the two Sm bands at 6 eV and near E_F correspond to the Sm^{3+} and Sm^{2+} configurations, respectively.

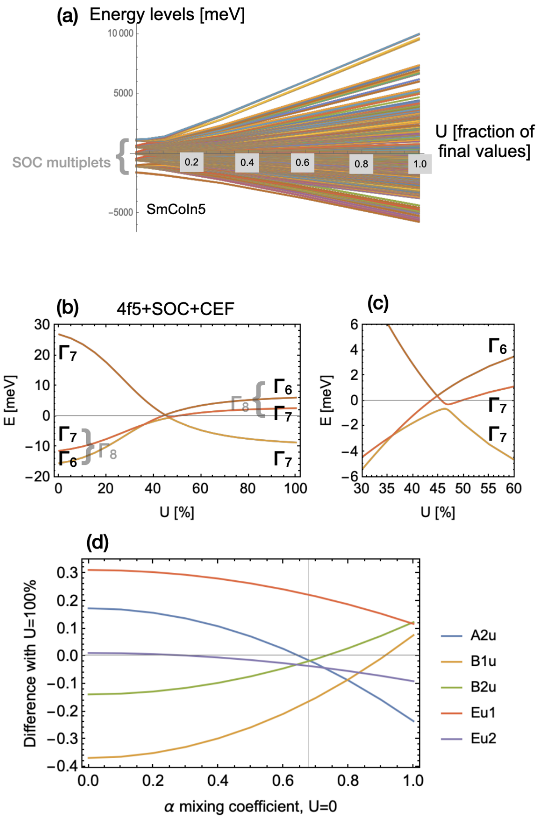

Supplementary Figure 1. Level scheme and effect of Coulomb repulsion on the ground state wavefunctions of Sm^{3+} in $SmCoIn_5$. (a) 2002 energy levels of Sm^{3+} as a function of the single-ion Coulomb interaction U, in units of a fraction of the final values used in this work. The six spin-orbit multiplets visible on the left side ($U = 0$) are significantly mixed and exhibit increased separation as U is increased. (b-c) Energy levels of the three Kramers doublet ground states, relative to the energy of the center of this group, plotted in percent change of U. In (b), the closeness of two levels over the entire range of U shows that they originate from the splitting of the Γ_8 octet of a higher-symmetry octahedral environment. In (c), the anti-crossing near $U \approx 47\%$ indicates that these levels both exhibit Γ_7 symmetry, thus fully identifying the symmetry of the three doublets. (d) Role of the Coulomb interaction U in the ground state of Sm^{3+} in $SmCoIn_5$, viewed from each symmetry-adapted irreducible representation (irrep) of the D_{4h} point group. Starting from the $J_z = {}^5\!/_2$ and $J_z = {}^3\!/_2$ spin-orbit eigenstates with $U = 0$, as the mixing parameter α between these states is varied, the irreps of the resulting composite wavevection are compared to their real values in $SmCoIn_5$ when U is included (measured in fractions of the real value).

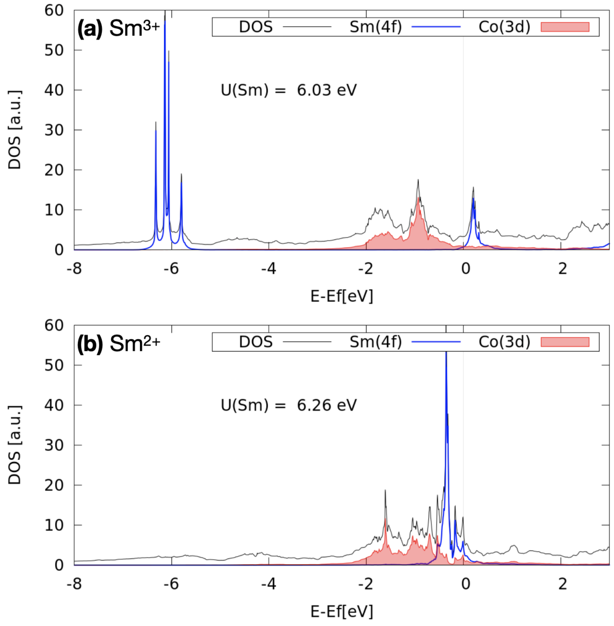

Supplementary Figure 2. Density of states of $SmCoIn_5$ from DFT calculations enforcing the (a) Sm^{3+} and (b) Sm^{2+} configurations.

Supplementary Note 3: Magnetization and inelastic neutron scattering experiments on $SmIrIn_5$

In Supplementary Figure 3, we show the raw inelastic neutron scattering data from EIGER using our $SmIrIn_5$ powder sample using the methods described in the main text, which we used to determine the crystal field parameters shown in Fig. 2(h) in the main text. The data were collected in a constant-Q method with fixed final neutron energy, at the six wavevectors shown in the figure. We assign two crystal field levels with black diamonds at energies of 17.5 and 20 meV, which is similar to the level scheme found for $SmCoIn_5$ from our XAS data. The feature shown as a black star disperses strongly with increasing $|Q|$ and therefore is not a crystal field level. For the two $|Q|$ positions with data taken to low energies, quasielastic scattering is visible with a similar linewidth found for the crystal field (diamonds) and dispersing feature (star), therefore we conclude that these measurements are close to the resolution limit of our experiment.

Using the energy levels determined from neutron scattering, the three independent crystal field parameters for Sm in $SmIrIn_5$ can be determined by comparing model calculations to measurements of the magnetic susceptibility. This methodology is similar to our approach to $SmCoIn_5$, in which we combined XAS experiments (main text Fig. 3) with magnetic susceptibility (Fig. 4). We determined that the best match to the magnetic susceptibility of $SmIrIn_5$ follows a similar behavior as with $SmCoIn_5$, where the susceptibility begins to deviate from the model below a delocalization temperature that is close to 100 K in $SmIrIn_5$. The magnetic susceptibility and corresponding model are shown in Supplementary Figure 4. The crystal field parameters are shown in Fig. 2 of the main text, and reproduce levels near 17.5 and 20 meV as we found from INS.

Supplementary Note 4: XAS experiments of $SmCoIn_5$

In Supplementary Figure 5, we show the geometry of the samples in the X-Treme endstation. In grazing incidence, where the experiments were carried out, the beam and mag-

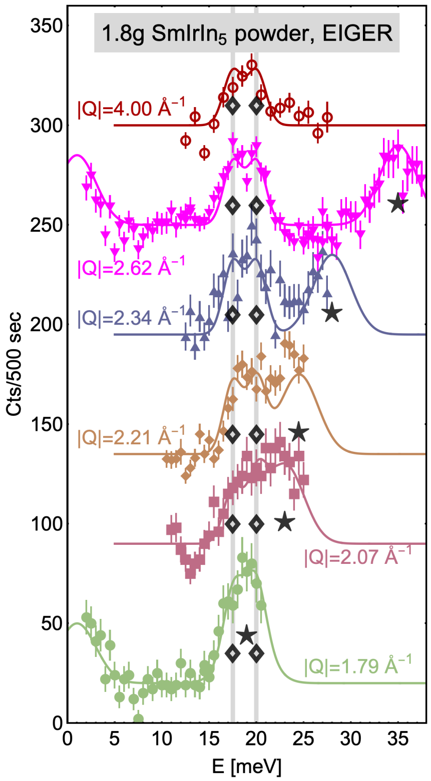

Supplementary Figure 3. Crystal field level scheme of $SmIrIn_5$ from inelastic neutron scattering (INS) measurements from EIGER. Each curve has been offset from zero to distinguish it, and error bars represent the standard counting error.

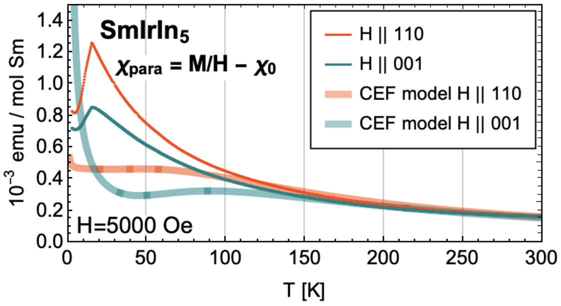

Supplementary Figure 4. Magnetization of $SmIrIn_5$ using the vibrating sample magnetometer, analogous to the experiments on $SmCoIn_5$ in Fig. 4 of the main text.

netic field direction is very close to the (101) crystallographic direction of the sample. In Supplementary Figure 6, we show the raw XMCD data as a function of temperature, collected at H=6.8 T along the (101) crystallographic direction (grazing incidence) as described in the main text. The XMCD signal is extremely large, on the order of 30% of the raw XAS spectra, giving us confidence that the sum rule analysis shown in Fig. 3(c) of the main text is accurate. To compute the integrals necessary to calculate the sum rule, the XAS scans were fitted to an inverse tangent function centered at each L edge, which was subtracted in order to get the total XAS integral. For the XMCD integrals, the left and right circularly polarized data was directly subtracted, leading to peaks at the two edges on top of a linear sloping background caused by the small drifts in the experimental conditions over time. After subtracting this linear background, the data were integrated and used to calculate the moments via the sum rule. In Supplementary Figure 7, we show the results of the XMCD analysis as a function of temperature, with the spin and orbital moments separately computed. The results assume a Co^{2+} ion (7 holes). Since there is no model fitting involved with the sum rule computation, a measure of the statistical error is not a good method to determine the accuracy of the sum rule analysis, and furthermore we do not know the appropriate choice of computing the statistical error in a way that is meaningful to the interpretation of the sum rule; therefore, no error bars are shown in Supplementary Figure 7 or in Fig. 4(c) of the main text. For different ionic states of Co, a scaling factor should be applied, which would not affect our main observation of an overall temperature trend. We also expect that an adjustment factor of about 10% should also be applied between the $L_{2,3}$ edges [4], somewhat changing the balance of the spin and orbital components of the magnetic moment, we did not apply this factor in order to showcase the raw data. Inclusion of this factor also does not affect our main conclusion.

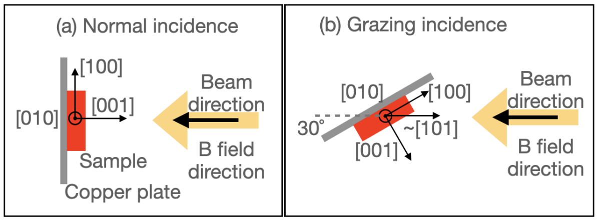

Supplementary Figure 5. Sketch of the XAS experiment geometry and sample configuration in (a) normal and (b) grazing incidence, where the experiments were carried out.

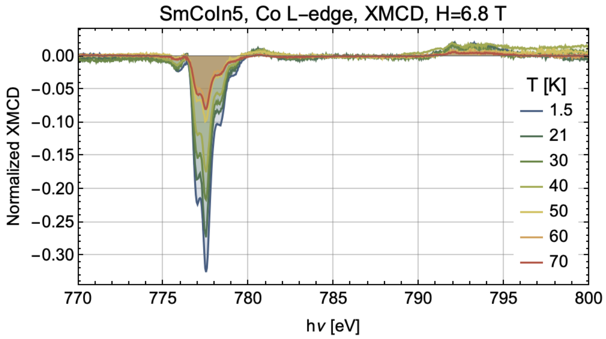

Supplementary Figure 6. Raw XMCD data from X-Treme, used for the sum rule analysis which generates the magnetic moment at the Co site, as shown in Fig. 4(c) of the main text.

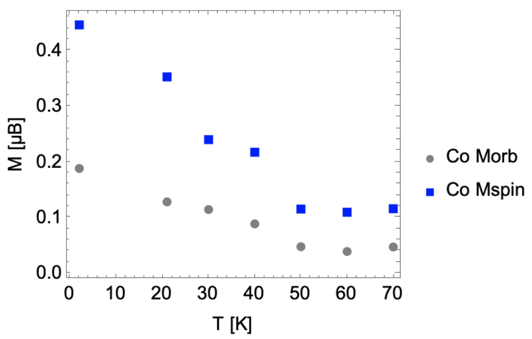

Supplementary Figure 7. Results of the XMCD analysis, showing spin and orbital magnetic moments. The results are added together in Fig. 4(c) of the main text.

Supplementary Note 5: Xray powder refinements for $SmCoIn_5$

Finally, in Supplementary Figure 8, we show a few of the powder patterns collected at MS using the methods described in the main text, displayed up to a scattering angle of $2\theta = 35$ degrees. The observed signal Yobs and calculated Ycal show an excellent match at all temperatures. We also found a small angle-dependent background exhibiting features up to about 40 counts, which we removed from the data using an iterative method. The remaining fitted background is shown in purple.

The iterative background removal procedure allowed us to reduce the error bars in the final refinements, determining more accurately the temperature at which the crossover temperature could be identified in Fig. 4(d-f) of the main text.

* david-william.tam@psi.ch
† michel.kenzelmann@psi.ch

Supplementary References

[1] Koster, G. F. Properties of the thirty-two point groups. (Cambridge, Mass., 1963).

[2] Fischer, G. & Herr, A. Representation of Energetical and Low-Field Magnetic Properties of J = 5/2 Rare Earth Ion States in Tetragonal Crystal Fields. physica status solidi (b) 141, 589–598 (1987).

[3] Willers, T. et al. Crystal-field and Kondo-scale investigations of Ce M In 5 (M = Co , Ir, and Rh): A combined x-ray absorption and inelastic neutron scattering study. Phys. Rev. B 81, 195114 (2010).

[4] Teramura, Y., Tanaka, A. & Jo, T. Effect of Coulomb Interaction on the X-Ray Magnetic Circular Dichroism Spin Sum Rule in 3 d Transition Elements. J. Phys. Soc. Jpn. **65**, 1053–1055 (1996).

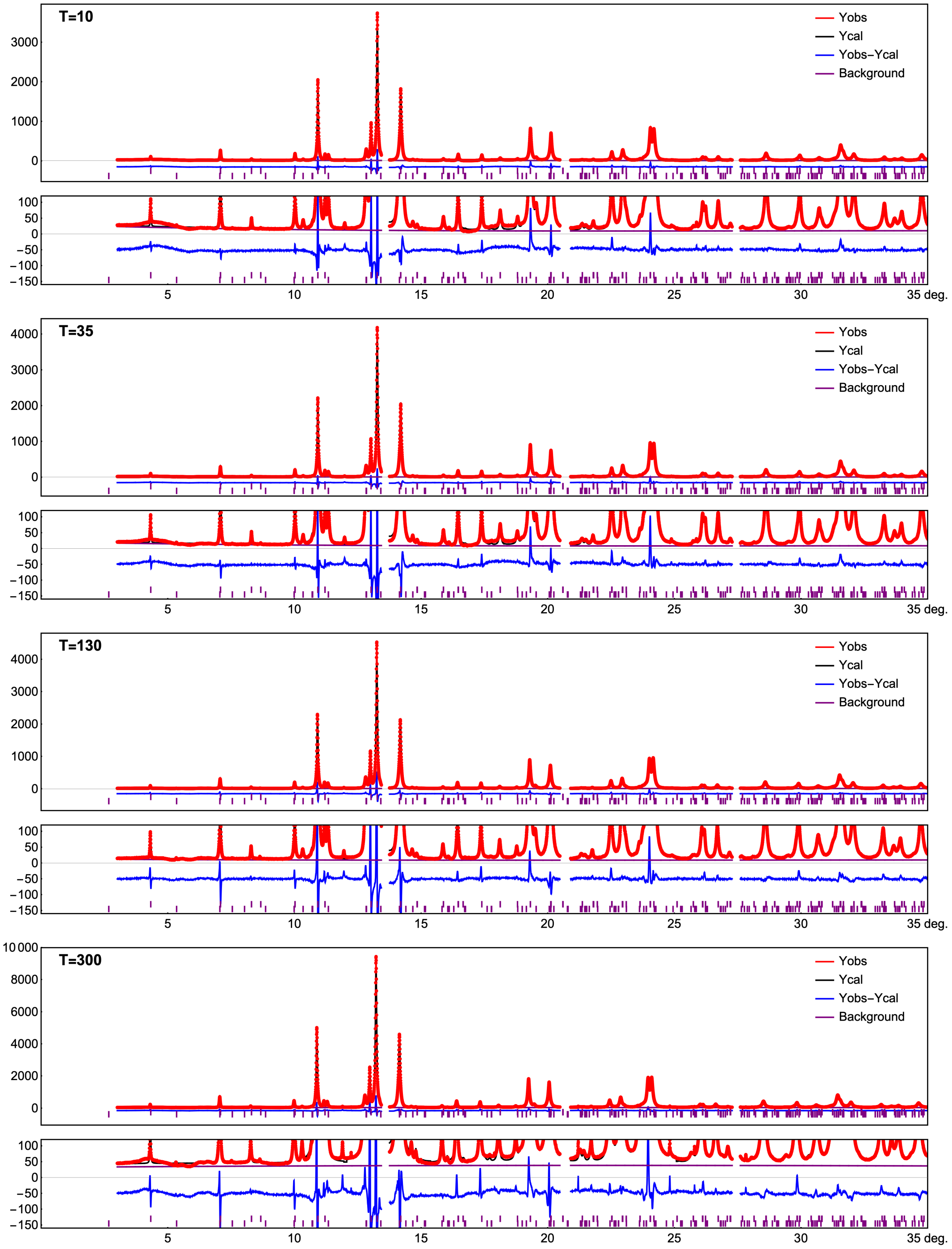

Supplementary Figure 8. Selected results of the powder refinement and Fullprof fits for data collected at MS. The upper row of peak positions corresponds to $SmCoIn_5$, while the lower row corresponds to peaks of the small Sm_2CoIn_8 impurity phase (2.9%). The blank regions are those containing small spurious peaks from the instrument.
